# Effect of bacillus subtilis strain Z15 secondary metabolites on immune function in mice

**DOI:** 10.1186/s12864-023-09313-5

**Published:** 2023-05-19

**Authors:** Xi-Yuan Cao, Reyihanguli Aimaier, Jun Yang, Jing Yang, Zhong-Yi Chen, Jing-Jing Zhao, Li Yin, Qi Zhang, Jia You, Hui Zhang, Hao-Ran Li, Jia-Yi Chen, Qing-Chen Mao, Li-Ping Yang, Fei Yu, He-Ping Zhao, Hui-Xin Zhao

**Affiliations:** 1 Xinjiang Key Laboratory of Special Species Conservation and Regulatory Biology, College of Life Science, Xinjiang Normal University, Urumqi, China; 2 Beijing Key Laboratory of Gene Resource and Molecular Development, College of Life Sciences, Beijing Normal University, Beijing, China

**Keywords:** *Bacillus subtilis*, Secondary metabolites, Animals, Transcriptome, Immune function

## Abstract

**Background:**

Previous studies have shown that secondary metabolites of *Bacillus subtilis* strain Z15 (BS-Z15) are effective in treating fungal infections in mice. To evaluate whether it also modulates immune function in mice to exert antifungal effects, we investigated the effect of BS-Z15 secondary metabolites on both the innate and adaptive immune functions of mice, and explored its molecular mechanism through blood transcriptome analysis.

**Results:**

The study showed that BS-Z15 secondary metabolites increased the number of monocytes and platelets in the blood, improved natural killer (NK) cell activity and phagocytosis of monocytes-macrophages, increased the conversion rate of lymphocytes in the spleen, the number of T lymphocytes and the antibody production capacity of mice, and increased the levels of Interferon gamma (IFN-γ), Interleukin-6 (IL-6), Immunoglobulin G (IgG) and Immunoglobulin M (IgM) in plasma. The blood transcriptome analysis revealed 608 differentially expressed genes following treatment with BS-Z15 secondary metabolites, all of which were significantly enriched in the Gene Ontology (GO) and Kyoto Encyclopedia of Genes and Genomes (KEGG) terms for immune-related entries and pathways such as Tumor Necrosis Factor (TNF) and Toll-like receptor (TLR) signaling pathways, and upregulated expression levels of immune-related genes such as Complement 1q B chain (C1qb), Complement 4B (C4b), Tetracyclin Resistant (TCR) and Regulatory Factor X, 5 (RFX5).

**Conclusions:**

BS-Z15 secondary metabolites were shown to enhance innate and adaptive immune function in mice, laying a theoretical foundation for its development and application in the field of immunity.

**Supplementary Information:**

The online version contains supplementary material available at 10.1186/s12864-023-09313-5.

## Introduction

Immune function refers to the ability of the immune system to recognize and destroy foreign invading foreign bodies (bacteria, fungi, viruses, etc.) and to remove senescent, damaged, dead and mutated cells as a mechanism of resistance to disease. A normal immune system prevents the invasion of pathogens and maintains normal functioning and physiological balance. However, disturbance of the immune system leads to impaired immune function and reduced defense against infection and cancer [1]. Furthermore, disease treatment options including surgery, organ transplantation, radiation therapy and the use of antibiotics can also disrupt immune function to varying degrees [2, 3], making it difficult to prevent and control fungal infections and other diseases. Therefore, enhancing immune function plays an important role in preventing the onset of disease and improving the outcome of clinical disease treatment.

Probiotics are microorganisms that are beneficial to the health of the host and stimulate the innate and adaptive immune systems [4, 5]. *Bacillus subtilis* is one of the main sources of probiotics and is classified as GRAS (Generally Recognized as Safe) by the US Food and Drug Administration (FDA). This organism has remarkable biosynthetic potential [6] and secretes a variety of secondary metabolites such as enzymes and lipopeptides that exhibit broad-spectrum antimicrobial [7], anticancer [8], antinematicidal activities [9], and immune activation in plants [10] and animals (11–13). Lipopeptides act as immunomodulators by interacting with pattern recognition receptors (PRRs), such as Toll-like receptors (TLRs), expressed on antigen-presenting cells (macrophages and dendritic cells) to activate immune functions [14]. *B. subtilis* secretes natural lipopeptides such as iturin, surfactin and mycosubtilin. WH1 fungin, a surfactin produced by *Bacillus amyloliquefaciens* WH1, induces a strong immune response to antigens without producing an adaptive immune response to itself [15]. As a result, *B. subtilis* and its secondary metabolites are widely used in agronomy, poultry farming and pharmaceutical production.

In previous studies, secondary metabolites of *B. subtilis* strain Z15 (BS-Z15) were found to be highly effective as a broad-spectrum antibacterial in *vitro* and could act as an elicitor to regulate systemic resistance in plants by inducing the immune response of plants [10]. Furthermore, in *vivo* studies demonstrated that BS-Z15 was safe and non-toxic in animals. However, it is not known whether BS-Z15 functions by antagonizing *Candida albicans* or by inducing an anti-fungal immunity. Therefore, in this study, we investigated the effects of BS-Z15 metabolites administered orally by gavaging on the innate immune function by testing blood physiological parameters, natural killer (NK) cell activity and phagocytosis of monocytes-macrophages, and adaptive immune function by testing the conversion rate of lymphocytes in the spleen, the number of T lymphocytes, the antibody production capacity and blood biochemical parameters. We also explored the molecular mechanisms through blood transcriptome analysis of the key genes and major pathways that regulate immune functions. This information will lay the foundation for the application of BS-Z15 and its secondary metabolites to improve immunity in animal husbandry and clinical applications.

## Materials and methods

### Preparation of BS-Z15 secondary metabolites

The BS-Z15 secondary metabolites were extracted according to the method described by Zhao et al. [16]. The BS-Z15 culture was centrifuged to obtain a sterile fermentation broth, acid precipitated overnight, centrifuged and resuspended in sterile water. The BS-Z15 secondary metabolites were then extracted with organic solvents, which were lyophilized and stored at -80 °C prior to use.

### Animal grouping and handling

Male and female immune systems vary considerably [17], so all the animals used in this experiment were male. After one week of acclimatization feeding, a first batch of animals comprising 120 specific pathogen-free (SPF) male Kunming mice (aged 5 weeks) were randomly divided into three groups: the control group received saline, the low-dose group received 30 mg/kg BS-Z15 secondary metabolites, and the high-dose group received 90 mg/kg BS-Z15 secondary metabolite; all treatments (0.4 ml/day per mouse) were administered orally by gavage at 9 am every morning for 40 days. Mice were given free access to food and water throughout the experiment. At the end of the treatment, 40 mice from the same treatment group were divided into three groups of 20, 10 and 10 mice, respectively, and various indexes were tested after different treatments. At 24 h after the last treatment, 20 mice in each group were fasted for 12 h and then anesthetized with 3 ml/kg 1.5% sodium barbiturate (Solarbio, China) by intraperitoneal injection before blood was collected from the main abdominal vein into collection tubes containing an anticoagulant and the spleen was removed aseptically. The blood and spleen were used for NK cell activity assays, splenic lymphocyte transformation, and blood physiology and biochemistry tests. At 24 h after the last treatment, 1 × 10 [9] sheep red blood cells (SRBC) (Solarbio, China) (200 µl) were administered intraperitoneally (total volume 1 ml) to 10 mice in each group at 9 am on four consecutive days. At 24 h after the last injection, the mice were fasted for 12 h and then anesthetized with 3 ml/kg 1.5% sodium barbiturate by intraperitoneal injection before the thickness of the right hindfoot metatarsal was measured using a precision Vernier caliper, blood was collected from the main abdominal vein and the spleen was aseptically removed for the detection of delayed metaplasia, antibody-producing cells and plasma hemolysin function. At 24 h after the last BS-Z15 secondary metabolite treatment, 10 µl/g India ink (Solarbio, China) (stock solution diluted 1:1,000 with saline) was injected into the tail vein of 10 mice per group for assays of monocyte-macrophage function.

After one week of acclimatization feeding, a second batch of animals comprising 18 SPF male Kunming mice (aged 5 weeks) was divided into two groups (n = 9 mice per group): the control group received saline and the treatment group received 90 mg/kg BS-Z15 secondary metabolite; all treatments (0.4 ml/day per mouse) were administered orally by gavage for 21 day. Blood was collected from the main abdominal vein after anesthesia by intraperitoneal injection of 3 ml/kg 1.5% sodium barbiturate. Samples from three mice in each group were pooled, with a total of six samples collected from both groups. The samples were snap frozen in liquid nitrogen for 30 min before storage at -80 °C prior to transcriptomic analysis.

### Effect of BS-Z15 secondary metabolites on blood physiological and biochemical parameters in mice

For evaluation of blood physiology parameters, whole blood samples collected into anticoagulation tubes were left to stand for 30 min before centrifugation at 1,000 ⋅*g* for 20 min. The plasma (upper layer) was aspirated and divided into aliquots that were snap frozen in liquid nitrogen for 30 min before storage at -80 °C. The samples were subsequently tested for four biochemical parameters (Immunoglobulin G; IgG, Immunoglobulin M; IgM, Interleukin-6; IL-6 and Interferon gamma; IFN-γ) according to the ELISA kit (LiankeBio, China) instructions.

### Effect of BS-Z15 secondary metabolites on mouse monocyte-macrophage function and NK cell activity

At 2 min (t1) and 10 min (t2) after the injection, blood samples (20 µl) were collected from the left and right medial canthus venous plexus into 2 ml 0.1% Na_2_CO_3_ solution. After light mixing, the optical density (OD) value was measured at 600 nm with a Multiskan Sky (Thermo, USA) and the OD value at t1 was recorded as lgOD1 and that at t2 as lgOD2, using 0.1% Na_2_CO_3_ solution as a blank control. Finally, the mice were euthanized before the liver and spleen were aseptically removed and weighed. The phagocytosis index α indicates the phagocytic capacity of mouse monocyte-macrophages with K as the uncorrected phagocytic index, which was calculated as follows [18]:

$$K=\frac{lgOD1-lgOD2}{t2-t1}$$

$$\begin{array}{l}Phagocytosis{\mkern 1mu} index{\mkern 1mu} \alpha = \\\frac{{Body{\mkern 1mu} weight}}{{Liver{\mkern 1mu} weight + Spleen{\mkern 1mu} weight}} \times \sqrt[3]{K}\end{array}$$

Fresh sterile spleen tissue collected from mice in different treatment groups was gently ground by adding 5 ml of Hanks’ balanced salt solution (HBSS) and filtered through a 200 mesh filter to obtain a single cell suspension. After centrifugation at 350 ×g for 5 min, the supernatant was discarded and the cell pellet was resuspended with 3 ml HBSS. The cells were then counted using a hemocytometer, and the centrifugation step was repeated to remove debris. The cells were resuspended in RPMI 1640 medium at 2 × 10^6^ cells/ml as effector cells, and. YAC-1 cells resuspended in RPMI 1640 medium at 2 × 10^5^ cells/ml as target cells. After seeding the above cells in 96-well plates with effector cells (2 × 10^5^ cells in 100 µl per well) and target cells (2 × 10^4^ cells in 100 µl per well) for the experimental well, target cells (2 × 10^4^ cells in 100 µl per well) and 100 µl 1640 medium for the target cell control well, and effector cells (2 × 10^5^ cells in 100 µl per well) added to 100 µl 1640 medium for the effector cell control group. Three replicate wells were included for each sample. After incubation at 37℃ for 2 h under 5% CO_2_, 10 µl MTT (5 mg/ml) was added to each well and the cells were incubated at 37 °C for a further 4 h. Finally, 150 µl dimethyl sulfoxide was added and plates were shaken for 10 min before measurement of the OD values at 570 nm with a Multiskan Sky multiplate spectrophotometer. NK cell activity was then calculated according to the following formula [19]:

$$\begin{array}{l}NK{\mkern 1mu} cell{\mkern 1mu} activity{\mkern 1mu} (\% ) = \\\left( {1 - \frac{{\exp erimental{\mkern 1mu} group{\mkern 1mu} OD - Effector{\mkern 1mu} cell{\mkern 1mu} control{\mkern 1mu} OD}}{{T\arg et{\mkern 1mu} cell{\mkern 1mu} control{\mkern 1mu} OD}}} \right) \times 100\% \end{array}$$

### Effects of BS-Z15 secondary metabolites on splenic lymphocyte transformation and delayed metaplasia in mice

#### Splenic lymphocyte transformation assay

Single cell suspensions of splenocytes from the different treatment groups of mice were prepared with HBSS as described in Sect. 2.5. Subsequently, 3 × 10^6^ splenocytes (1 ml) suspension was added to the control and experimental tubes. After the addition of 75 µl ConA solution to the experimental tubes, the cells were incubated for 72 h at 37℃ under 5% CO_2_. The supernatant was removed by centrifugation at 300 ⋅*g* for 5 min before the addition of 1 ml RPMI 1640 basal medium and 50 µl MTT (5 mg/ml). The cells were then incubated for 4 h at 37 °C before 1 ml acidic isopropanol was added. The OD at 570 nm with a Multiskan Sky multiplate spectrophotometer was measured after ultrasonic shaking to dissolve the purple crystals.

#### Evaluation of delayed metaplasia

After experimental treatment, 1 × 10^9^ RBC/ml SRBC (200 µl) was administered intraperitoneally at 9 am for four consecutive days. The thickness of the right hindfoot and plantar area was then measured with a precision Vernier caliper before 20 µl 1 × 10^10^ RBC/ml SRBC was injected subcutaneously at the measurement sites. After 24 h, measurements at these sites were repeated three times by the same person and the difference between the anterior and posterior foot and plantar area was calculated to represent the degree of swelling.

### Effect of BS-Z15 secondary metabolites on antibody-producing cells and plasma hemolysin function in mice

#### Antibody-producing cell assay

Single cell suspensions were prepared from sterile mouse spleen tissue in HBSS. Subsequently, 2 × 10^7^ splenocytes (1 ml) suspension was added to the control and experimental tubes. After the addition of 1 ml HBSS to the control tubes and 1 ml each of 1 × 10^9^ RBC/ml SRBC and 10-fold diluted complement to the experimental tubes, the cells were incubated for 1 h at 37 °C in a water bath, and then centrifuged at 1400 ×g for 5 min. The supernatant was collected and the OD value at 413 nm was measured using a spectrophotometer (Metash, China).

#### Serum hemolysin function assay

Blood was collected intraperitoneally from mice using a vacuum blood collection tube without anticoagulant. After standing for 30 min at room temperature, the serum was separated by centrifugation at 1,000 ⋅*g* for 20 min, and diluted 1:100 with 1⋅ SA buffer. After 100 µl diluted serum was added to each sample tube, and 100 µl 1⋅ SA buffer was added to each blank control tube, 50 µl 1⋅10^8^ RBC/ml SRBC and 100 µl complement (diluted 1:8 with 1⋅ SA buffer) were added to all tubes. After incubation for 30 min in a water bath at 37 °C, the tubes were centrifuged for 10 min at 350 ×g and 50 µl supernatant from each tube was added to 96-well culture plates. Followed by 150 µl Du’s reagent. After adding 25 µl 1⋅10^8^ RBC/ml SRBC to half of the hemolysis assay wells, 175 µl Du’s reagent was added. The plates were mixed thoroughly with a shaker and left at room temperature for 10 min before the OD value of each well at 540 nm was measured with a Multiskan Sky multiplate spectrophotometer.

### Transcriptomic analysis

Total RNA was extracted from blood samples using TRIzol® reagent. The RNA integrity number (RIN) was determined using an Agilent 2100 Nanodrop and quantified using an ND-2000 (Nanodrop Technologies, USA). Samples with RIN ≥ 9, OD260 nm/280 nm values > 1.9 and OD260 nm/230 nm values > 2.1, respectively, were used to construct sequencing libraries using the Illumina TruSeq™ RNA Sample Preparation Kit (Illumina, San Diego, CA, USA) using standard procedures. The libraries were sequenced by Majorbio Biotech (Shanghai, China) using the Illumina NovaSeq 6000 platform. The sequencing data were filtered and all subsequent analyses were based on Cleandata.

Expression levels of genes and transcripts were quantified separately using RSEM (http://deweylab.github.io/RSEM/) [20]. Differences in gene expression between treatment groups were analyzed by edgeR and DESeq2, and functions were annotated by comparing unigenes with multiple databases, including Gene Ontology (GO, http://www.geneontology.org), Kyoto Encyclopedia of Genes and Genomes (KEGG, http://www.genome.jp/kegg) [21–23], NCBI Non-Redundant (NR, http://www.ncbi.nlm.nih.gov), Swiss-Prot (http://www.expasy.ch/sprot), Pfam (http://pfam.xfam.org), and Protein Direct Homology Group Cluster (EggNOG, http://eggnogdb.embl.de/#/app/home). GO and KEGG enrichment analyses of genes in the gene set were performed using Goatools software and R scripts [24], respectively.

### Real-time fluorescence quantitative PCR validation of transcriptomic data

Six genes associated with immunity were selected from the list of DEGs to validate the transcriptome data. The primers of the selected genes were designed by NCBI and synthesized by Sangon Biotech (Shanghai); the sequences are listed in Table S1. Real-time fluorescence quantitative PCR (qPCR) was performed using SuperReal PreMix Plus (SYBR Green) (TIANGEN, USA) according to the three-step method in the instructions with three biological replicates and three technical replicates, Temperature program: [1] 95 °C for 15 min for 1 cycle, [2] 95 °C for 10 s, [3] 50–60 °C for 20 s, [4] 72 °C for 30 s, [2] [3] [4] repeat for 40 cycles. β-actin was used as the reference gene. Relative gene expression levels were calculated using the 2^−ΔΔCt^ method, with results in the form of relative Log2 fold changes as described by Bustin et al. [25].

### Statistical analysis

Experimental data were expressed as the mean ± standard deviation (SD). One-way ANOVA was performed with SPSS 20, and comparisons between groups were made using Duncan’s multiple range test, with *P* < 0.05 set as the threshold for statistical significance. Graphs were created using Sigma Plot and Adobe illustrator. Hierarchical clustering and heat maps of transcriptomic data were generated using Meguiar’s platform, GraphPad prism 12.5 and Adobe illustrator.

## Results and analysis

### Effect of BS-Z15 secondary metabolites on innate immune function in mice

**Fig. 1:**
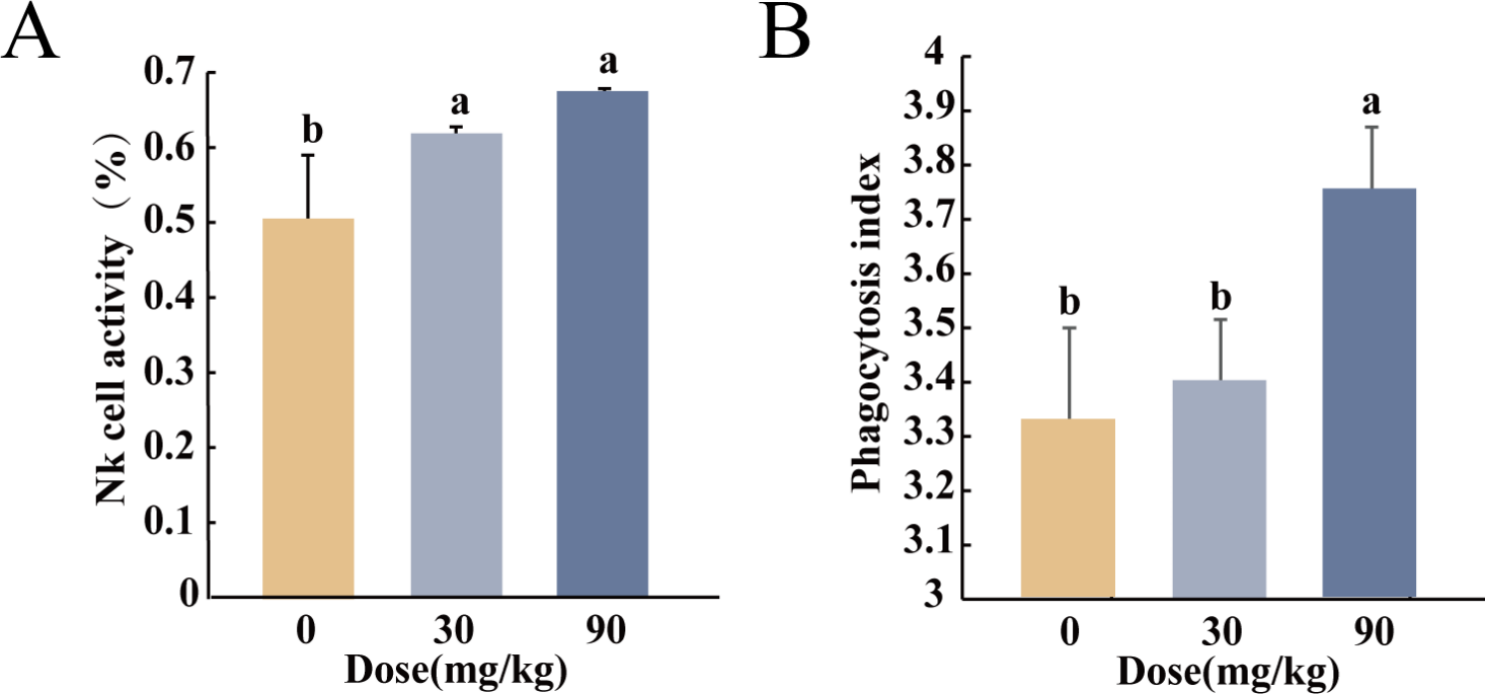
Effects of BS-Z15 secondary metabolites on innate immunity (**A**) (**B**)in mice. (**A**) Effects of BS-Z15 secondary metabolites on mouse NK cell activity (n = 8–11). (**B**) Effects of BS-Z15 secondary metabolites on phagocytosis of mouse monocyte-macrophages (n = 3)

Compared with the control group, NK cell activity was significantly enhanced by administration of BS-Z15 secondary metabolites in both the low and high-dose groups, with increases of 24.99% (*P* < 0.05) and 41.81% (*P* < 0.05) respectively, and no significant difference (*P >* 0.05) between the two groups (Fig. 1A).

Compared with the control group, there was no significant change in carbon contour phagocytosis index in the low-dose group (*P >* 0.05). However, a significant increase of 12.77% was observed in the high-dose group (*P* < 0.05) (Fig. 1B), indicating that BS-Z15 secondary metabolite treatment enhanced the ability of NK cells to kill target cells and phagocytosis of monocytes-macrophages in mice, thereby improving innate immune function in mice.

#### Effect of BS-Z15 secondary metabolites on blood physiological parameters in mice

Compared to the control group, the number of monocytes in the blood of mice in both the low and high-dose groups increased significantly, by 6.45% (*P* < 0.05) and 12.9% (*P* < 0.05), respectively, (Table 1). In addition, BS-Z15 secondary metabolites significantly increased the monocyte and platelet counts in the blood of mice, with increases of 2.7% (*P* < 0.05) and 10.59% (*P* < 0.05) observed in the low and high-dose groups, respectively.

**Table 1:** Effect of BS-Z15 metabolites on blood physiological parameters in mice**(*x ± s***, ***n =*** **10)**

Parameters	Unit	Projects	Gavage dose		
			0 mg/kg	30 mg/kg	90 mg/kg
WBC	10^9^/L	Leukocyte	2.209	2.147	2.255
Neu	10^9^/L	Neutrophil count	0.937	0.935	0.937
Lym	10^9^/L	lymphocyte count	1.166142857	1.132857	1.225
Mon	10^9^/L	monocyte count	0.031	0.033^*^	0.035^*^
Eos	10^9^/L	eosinophil count	0.071	0.044	0.053
Bas	10^9^/L	Basophil count	0.0042	0.0042	0.005
RBC	10^12^/L	number of red blood cells	8.52	8.656	8.646
HGB	g/L	hemoglobin	136.2857143	132.14	132.875
MCV	fL	mean red blood cell volume	45.47	44.77	44.53
MCH	pg	mean corpuscular hemoglobin	15.82	16.38	16.47
MCHC	g/L	mean hemoglobin concentration	368.4285714	365.7143	369.875
PLT	10^9^/L	Platelet count	963.27	989.25^*^	1065.33^*^

Note: Compared with control, *P < 0.05;**P < 0.01.

### Effect of BS-Z15 secondary metabolites on adaptive immunity

#### Effect of BS-Z15 secondary metabolites on cellular immune function in mice

**Fig. 2:**
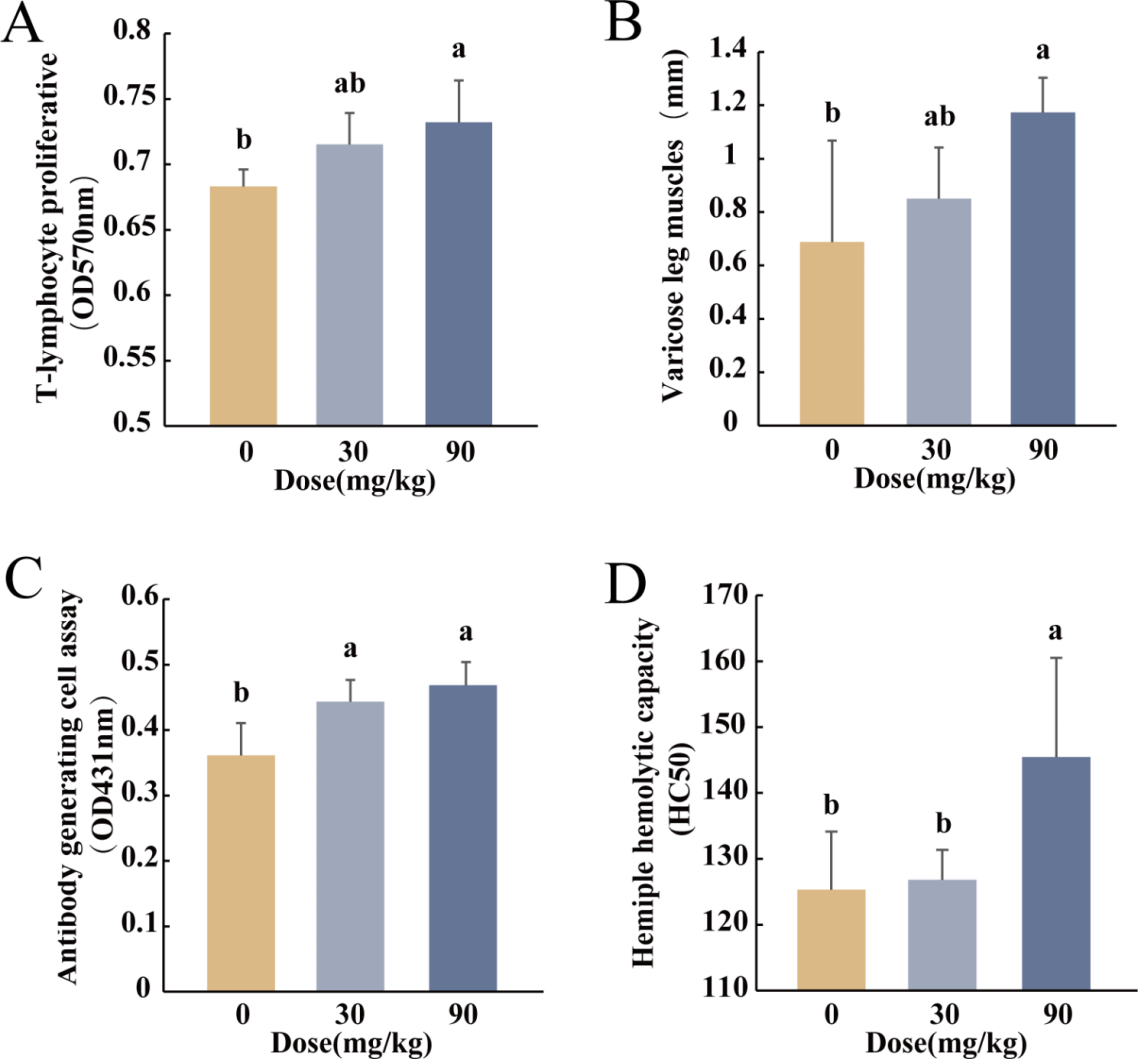
Effects of BS-Z15 secondary metabolites on cellular immunity (**A**) (**B**), and humoral immunity (**C**) (**D**)in mice. (**A**) Effect of BS-Z15 secondary metabolites on T-lymphocyte proliferative in mice (n = 4). (**B**) Effects of BS-Z15 secondary metabolites on delayed-type hypersensitivity in mice (n = 8–9). (**C**) Effect of BS-Z15 secondary metabolites on the activity of mouse antibody-producing cells (n = 10). (**D**) Effects of BS-Z15 secondary metabolites on serum hemolysin levels in mouse cells (n = 10). Bar graphs are expressed as mean ± SEM. P-values of less than 0.05 were considered statistically significant, different letters were considered significant differences, and the same letters were not significantly different

Compared to the control group, there was a dose-dependent increase in T cell conversion in the BS-Z15 secondary metabolite treated groups, with a significant increase of 7.17% observed in the high-dose group (*P* < 0.05), although the 4.7% increase in the low-dose group (*P* > 0.05) did not reach the level of statistical significance (Fig. 2A).

Compared to the control group, the delayed type hypersensitivity-mediated swelling of the left hind foot and metatarsal area of mice was in by 23.54% in the low-dose group (*P* < 0.05) and by 70.54% in the high-dose group (*P* < 0.05) (Fig. 2B), indicating that treatment with BS-Z15 secondary metabolites enhanced cellular immunity in mice.

### Effect of BS-Z15 secondary metabolites on humoral immune function in mice

Compared to the control group, the ability of antibody-forming cells to produce antibodies in the spleen was significantly increased in both the low (22.77%, *P* > 0.05) and high (29.79%, *P* < 0.05) groups (Fig. 2C), with no significant difference between the two groups.

Compared to the control group, there was no significant difference in the hemolytic function of the plasma of the low-dose group (*P* > 0.05), although there was a significant increase of 16.06% in the high-dose group (*P* < 0.05) (Fig. 2D), indicating that BS-Z15 secondary metabolite treatment improved antibody production and enhanced humoral immune function in mice.

#### Effect of BS-Z15 secondary metabolites on blood biochemical parameters in mice

**Fig. 3:**
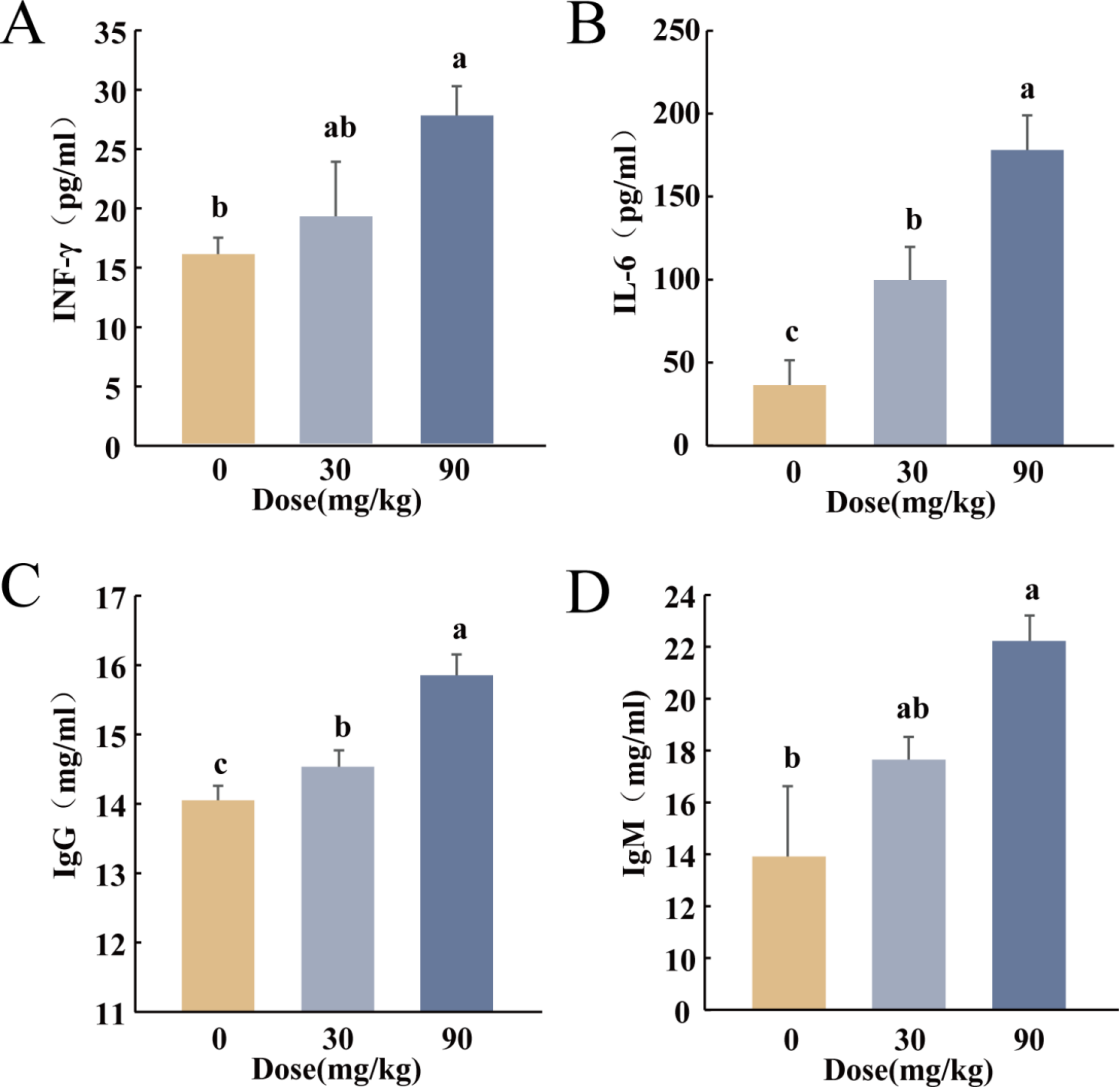
Effects of BS-Z15 secondary metabolites on immune cytokine (**A**) (**B**) and immunoglobulin (**C**) (**D**) levels in mouse plasma. (**A**) IFN-γ (**B**) IL-6 (**C**) IgG (**D**) IgM (n = 10). Bar graphs are expressed as mean ± SEM. P-values of less than 0.05 were considered statistically significant, different letters were considered significant differences, and the same letters were not significantly different

Figure 3 shows the effect of BS-Z15 secondary metabolites treatment on blood biochemical parameters in mice. Compared to the control group, the plasma IFN-γ content was significantly increased by 73.33% in the high-dose group (*P* < 0.05), while the growth rate of 20% in the low-dose group (*P* > 0.05) was not significant (Fig. 3A). The plasma IL-6 levels were also significantly increased in both the low (174.2%, *P* < 0.05) and high (389.26%, *P* < 0.05) dose groups (Fig. 3B). Furthermore, the plasma IgG content increased significantly in both the low (3.44%, *P* < 0.05) and high (12.85%, *P* < 0.05) dose groups (Fig. 3C). While the plasma IgM content increased by 25.57% in the low-dose group (*P* > 0.05), the levels increased significantly by 58.84% in the high-dose group (*P* < 0.05) (Fig. 3D). These results showed that 90 mg/kg BS-Z15 secondary metabolite treatment increased the levels of IFN-γ, IL-6, IgG and IgM in the blood of mice.

### Transcriptome sequencing-based analysis of the effects of BS-Z15 secondary metabolites treatment on the expression of immune-related genes in mice

#### Transcriptome data quality control and differentially expressed gene analysis

The raw sequencing data from each sample were assessed for sequencing-related quality using Fastp software [26], resulting in 53.77 million and 47.35 million high-quality pure reads (clean reads) for the control and treatment groups, respectively, for assembly and further downstream analysis. As shown in Table 2, all samples exceeded 98.75% for Q20 and 96.13% for Q30, with a good distribution of GC content, indicating that the RNA sequence data for all samples were of high quality and suitable for use in subsequent bioinformatics analysis.

**Table 2:** RNA SEQ data information of mouse blood samples with or without BS-Z15 secondary metabolites administration

Sample	Raw reads	Clean reads	Error rate(%)	Q20(%)	Q30(%)	GC content(%)
CK1	60,580,112	59,941,094	0.0229	98.83	96.38	56.26
CK2	45,970,274	45,528,578	0.0231	98.75	96.13	56.23
CK3	56,528,142	55,867,780	0.0228	98.88	96.46	56.25
MEs1	43,483,116	42,681,114	0.023	98.79	96.3	55.86
MEs2	51,778,572	51,222,910	0.0229	98.83	96.31	56.58
MEs3	48,660,452	48,158,388	0.0229	98.85	96.37	56.41

Note: CK1, CK2 and CK3 are blood sequencing samples of control group mice. MEs1, MEs2 and MEs3 are blood sequencing samples of treatment group mice

**Fig. 4:**
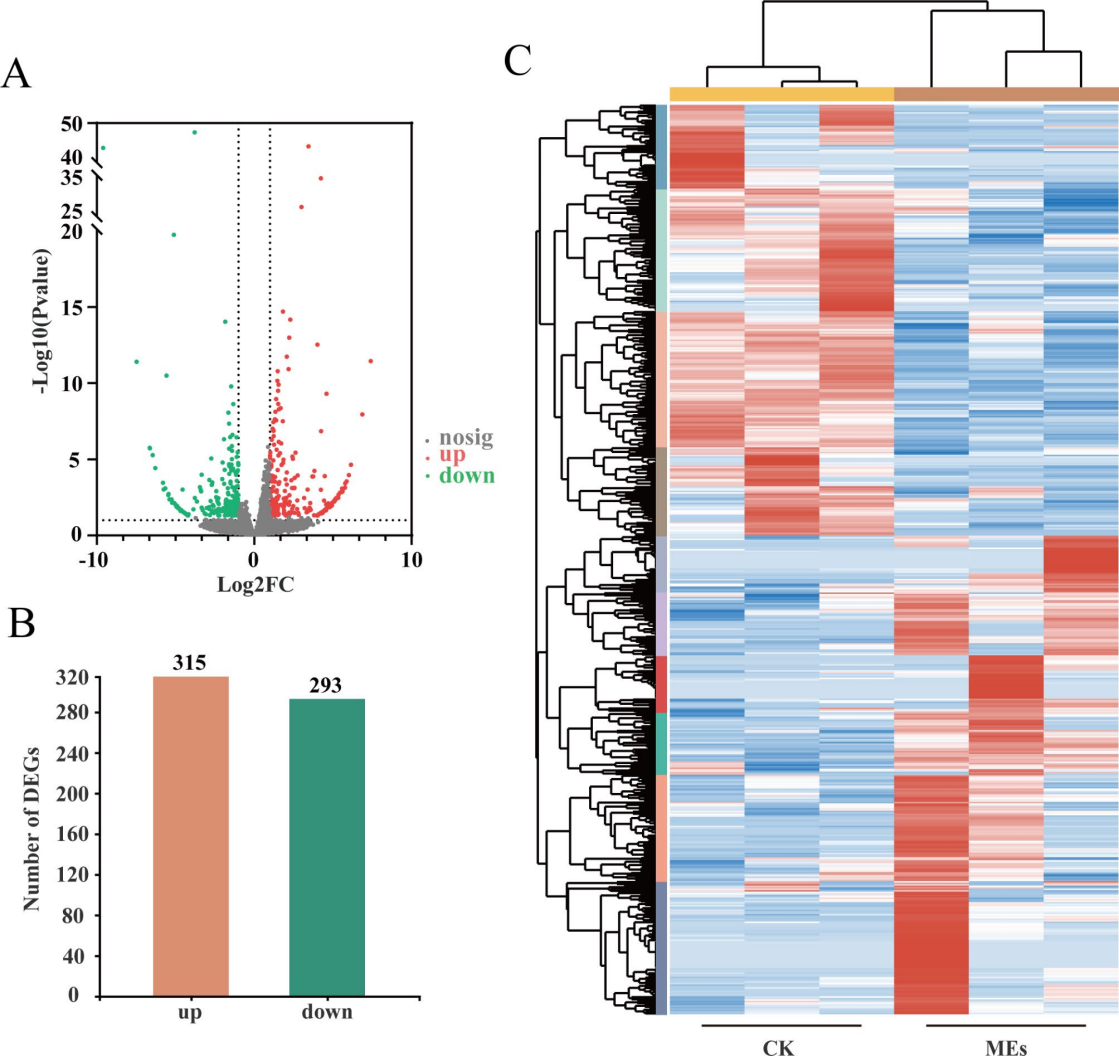
Analysis results of differentially expressed genes. (**A**) Distribution of DEGs volcanoes. X-axis is the display of log2 fold change, Y-axis is -log10Pvalue; Red represents upregulation gene, green represents downregulation gene, gray represents non differentiation gene. (**B**) Statistical chart of the number of DEGs. The abscissa is DEGs, and the ordinate is the number of difference genes; Red represents upregulation gene, green represents downregulation gene. (**C**) Clustering diagram of different groups. Red indicates high expression of gene and blue indicates low expression of gene

Variation in gene length and total read distribution in gene expression levels was eliminated by FPKM calculations. Differentially expressed genes were screened using an absolute value of log2 fold change ≥ 1 (i.e., the change in gene expression in the treated groups compared with the control group was more than 2-fold, with a statistical significance threshold of *P* < 0.05). A volcano plot showing the distribution of DEGs is shown in Fig. 4A. As shown in Figs. 4B and 608 genes showed significant differential expression in the blood of the BS-Z15 secondary metabolites treated group compared to the control group. Of these DEGs, 315 were upregulated and 293 were downregulated. A heat map of cluster analysis of these DEGs is shown in Fig. 4C.

#### GO database analysis of differentially expressed genes

**Fig. 5:**
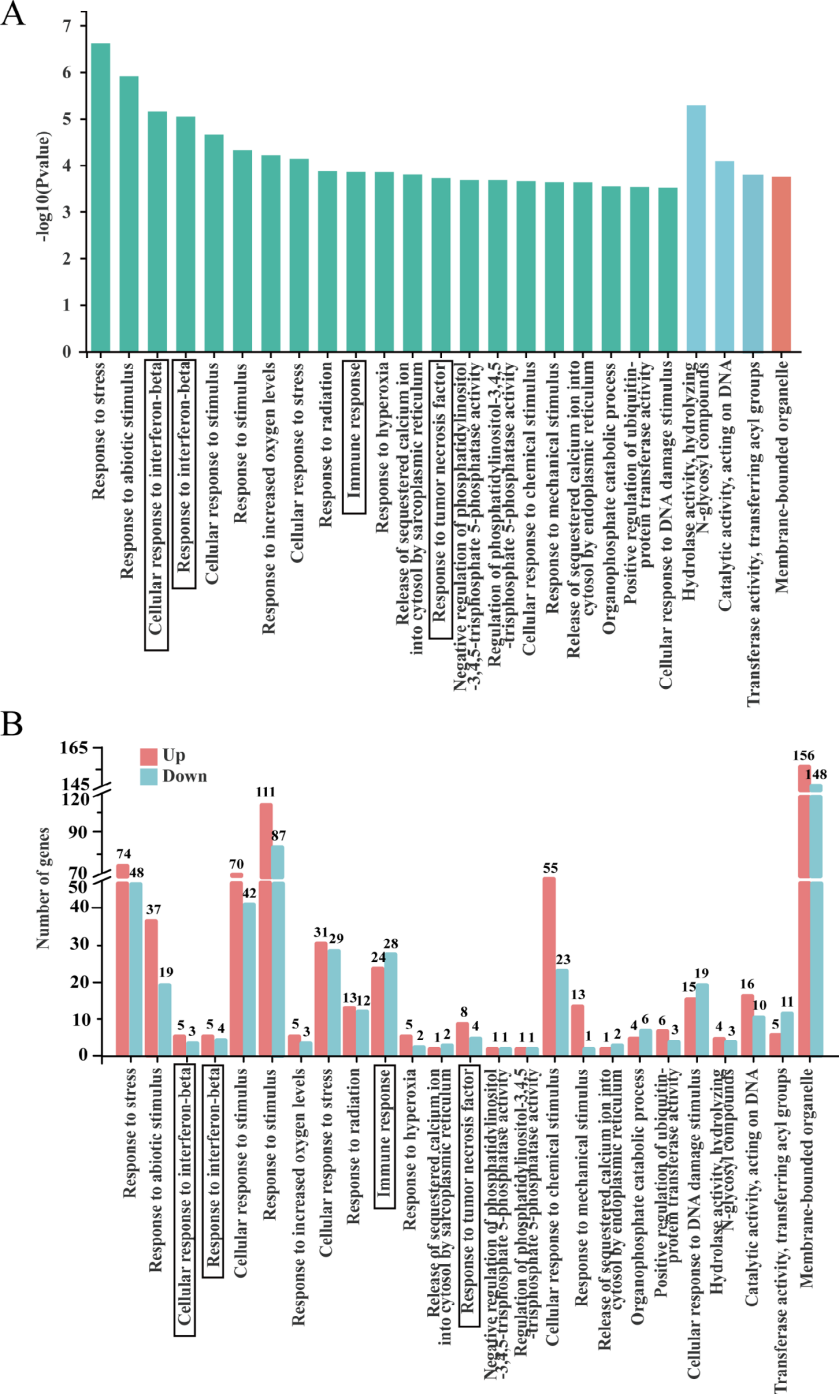
DEGs were analyzed by GO enrichment. (**A**) GO enrichment analysis of DEGs. The horizontal axis represents -log10 P-value, and the vertical axis represents the GO term. Red represents cellular component (CC), blue represents molecular function (MF), green represents biological process (BP). (**B**) Comparison of upregulation and downregulation number of gene at GO term. Red indicates up regulation of GO term enriched by DEGs, blue indicates down regulation of GO term enriched by DEGs, the horizontal axis is the GO term name, and the vertical axis is the number of gene of corresponding terms. Immune-related items are marked with black boxes

To illustrate the functions of these DEGs, GO enrichment analysis was performed in terms of biological processes (BP), cellular component (CC) and molecular function (MF). In total, 866 GO terms were located in the GO enrichment analysis, with the top 25 GO terms in terms of abundance shown in Fig. 5A. Sixteen of the GO terms were located in the BP category, three in MF and one in CC. The main immune-related terms were cellular response to IFN-β, response to IFN-β, immune response, and response to tumor necrosis factor. The number of differentially up- and downregulated genes in the top twenty-five GO terms in terms of abundance are shown in Fig. 5B. In terms of differential gene expression trends, five DEGs were upregulated and three were downregulated in cellular response to IFN-β, five DEGs were upregulated and four downregulated in response to IFN-β, 24 DEGS were upregulated and 28 were downregulated in immune response, and eight DEGs were up-regulated and four were down-regulated in response to tumor necrosis factor. These findings suggested that BS-Z15 secondary metabolites have immunomodulatory effects.

#### KEGG analysis pathway enrichment of the differentially expressed genes

**Fig. 6:**
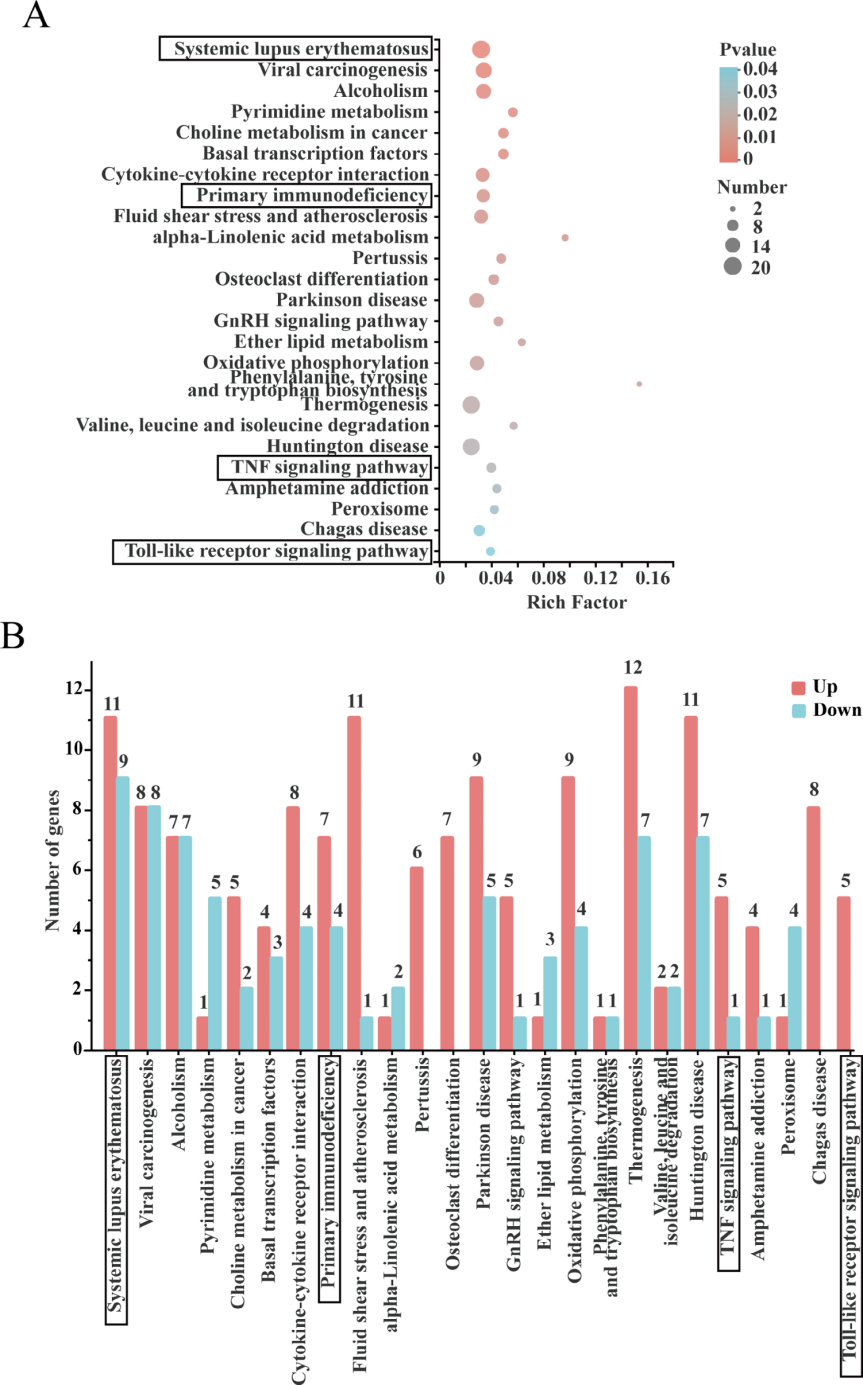
DEGs were analyzed by KEGG enrichment. (**A**) KEGG enrichment analysis of DEGs. The horizontal axis represents Rich Factor, and the vertical axis represents the KEGG pathway. The size of the point represents the number of genes in this KEGG pathway, and the color of the point corresponds to different P-value ranges. (**B**) Comparison of upregulation and downregulation number of gene at KEGG pathway. Red indicates up regulation of KEGG pathway enriched by DEGs, blue indicates down regulation of KEGG pathway enriched by DEGs, the horizontal axis is the KEGG pathway name, and the vertical axis is the number of gene of corresponding pathways. Immune-related pathways are marked with black boxes

KEGG pathway enrichment analysis revealed involvement of the significant DEGs in a total of 276 metabolic pathways. Among the top 25 metabolic pathways shown in Fig. 6A, systemic lupus erythematosus, viral carcinogenesis and alcoholism pathways were the most significantly expressed. In addition, immune pathways such as primary immunodeficiency, TNF signaling pathway and TLR signaling pathway, were significantly differentially expressed.

The number of DEGs upregulated and downregulated in the top 25 KEGG pathways in terms of abundance are shown in Fig. 6B. In terms of differential gene expression trends, 11 DEGs were upregulated and nine were downregulated in systemic lupus erythematosus, seven DEGs were upregulated and four were downregulated in primary immunodeficiency, five DEGs were upregulated and one was downregulated in TNF signaling pathway, and five were upregulated and none were downregulated in Toll-like receptor signaling pathway. The DEGs in the Toll-like receptor signaling pathway included Gm17809, encoding IKKα, Gm49320, encoding MMK7, and the transcription factors Jun and Fos. The TNF signaling pathway negative regulatory protein synthesis death domain silencer (SODD) Bag4, and the chemokine Ccl3. Thus, These findings provided further evidence that BS-Z15 secondary metabolites have immunomodulatory effects.

#### Immune-related gene analysis and real-time fluorescence quantitative PCR validation

The GO and KEGG enrichment analyses of immune-related entries and pathways revealed that 74 immune-related genes were differentially expressed in the treated group compared to the control group, including 39 upregulated DEGs and 35 downregulated DEGs (Fig. 3C). Upregulated genes included those encoding interleukin-7 (Il7) and complement components (C1qb, C4b), which are widely involved in non-specific and specific immune responses, and those encoding transmembrane protein 98 (Tmem98), the T cell antigen receptor (Trav16d-dv11), and the transcription factor Regulatory Factor X, 5 (RFX5) (B230398E01Rik), as well as transforming factor-related genes (Tgfb2, Fstl3), which regulate the innate immune response. The main genes downregulated were those encoding chemokine (Ccr5), CD40 ligand (CD40lg), and histone H4 (Gm20634). These findings indicated that BS-Z15 secondary metabolite treatment modulated the expression of immune-related genes in mice.

**Fig. 7:**
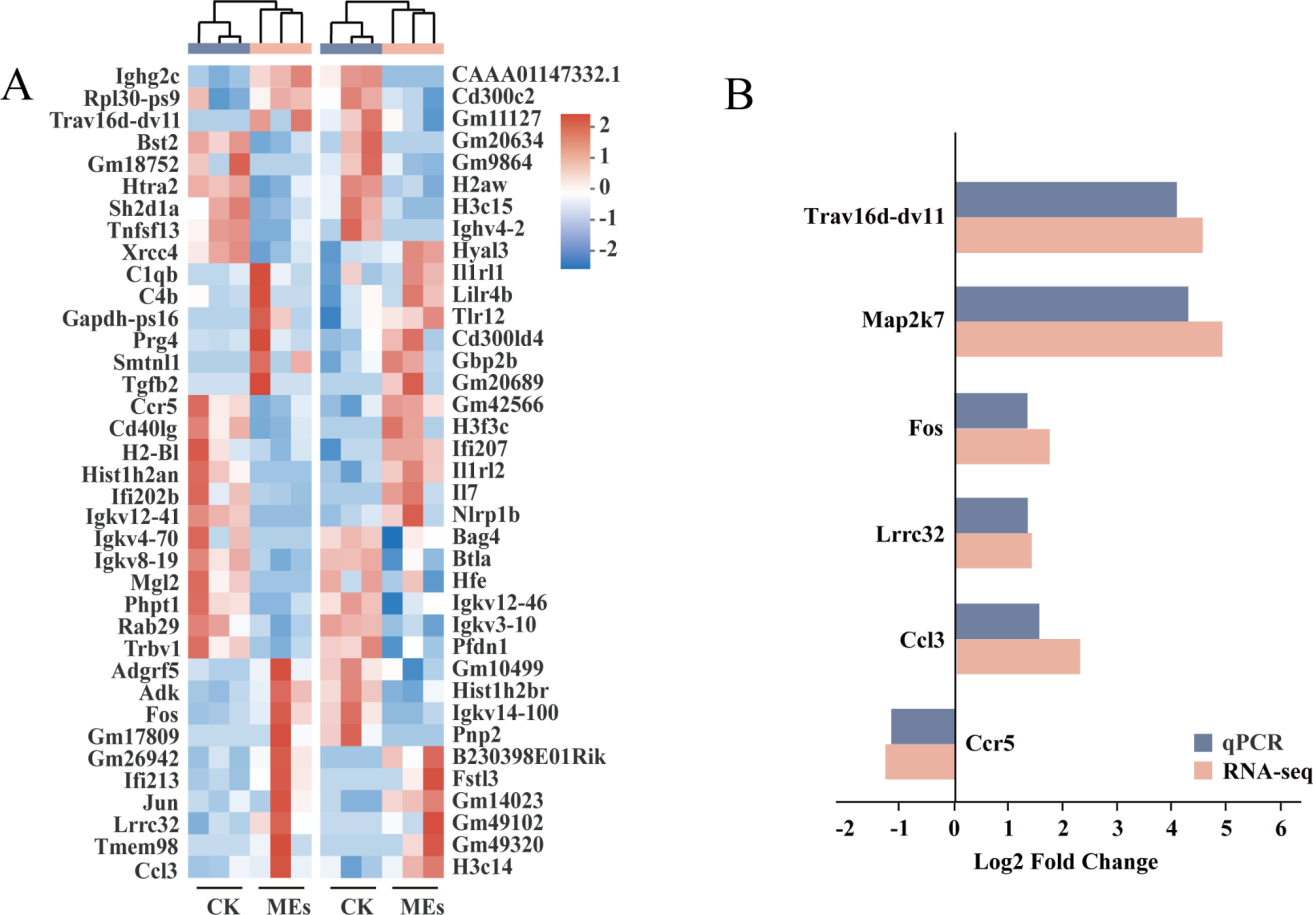
(**A**) Clustering heatmap of immune-related DEGs sorted by high Log FC. Red indicates high expression of gene and blue indicates low expression of gene.(**B**) Log2 fold change of 6 immune-related DEGs screened by qPCR and transcriptome. The vertical axis represents log2 fold change and the horizontal axis represents the name of genes, black represents qPCR data and grey represents transcriptome data results

To verify the reliability of the RNA-seq transcriptomic data, six immune-related genes (Trav16d-dv11, MKK7, Fos, Lrrc32, Ccl3, and Ccr5) were selected for qPCR validation, with β-actin as an internal reference gene. As shown in Fig. 7, Trav16d-dv11, MKK7, Fos, Lrrc32 and Ccl3 were upregulated after BS-Z15 secondary metabolite treatment, while Ccr5 was downregulated. The qPCR validation was consistent with the expression patterns of the six DEGs obtained by RNA-seq, indicating that the RNA-seq results reliably reflected the gene expression trends.

## Discussion

**Fig. 8:**
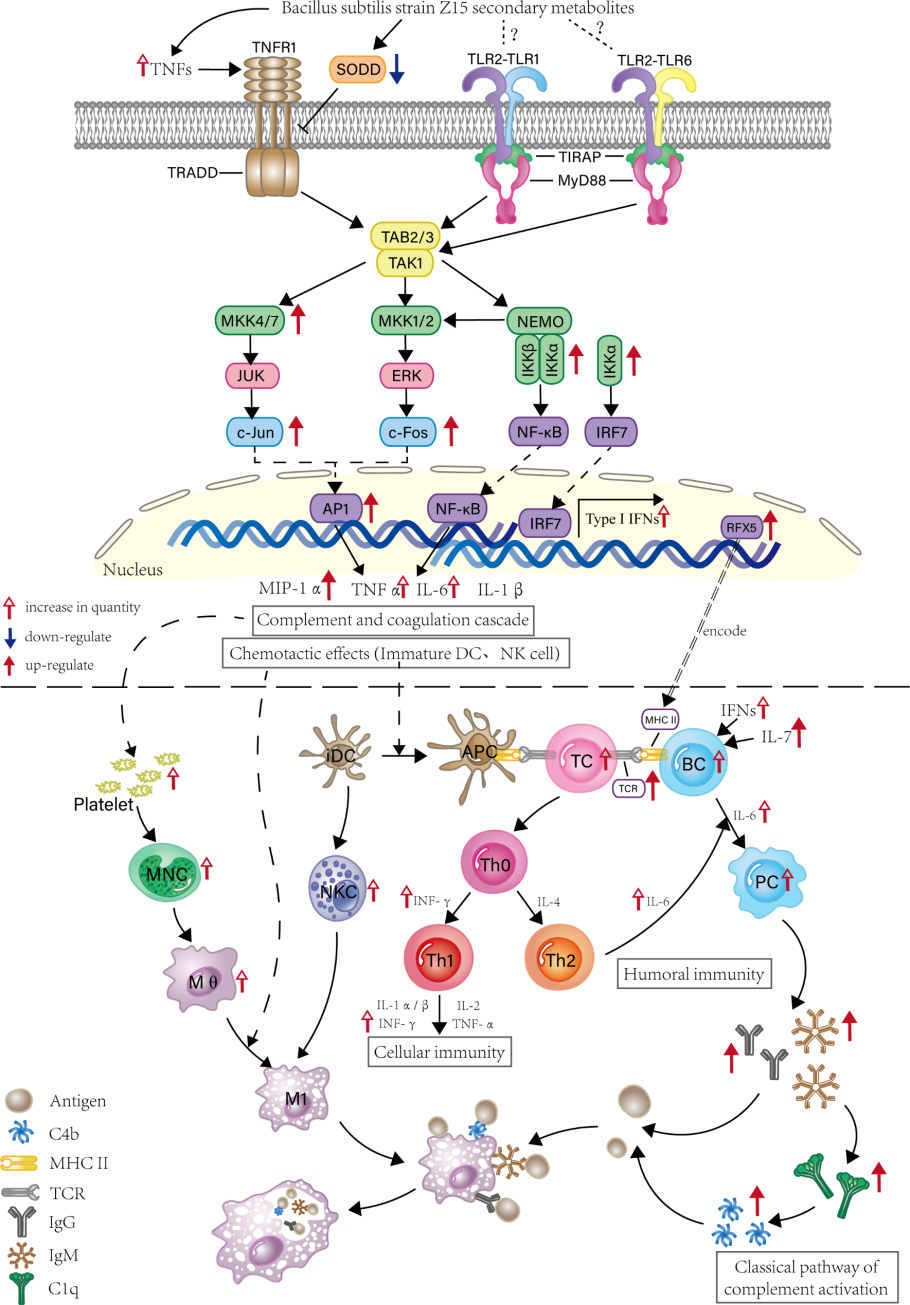
Mechanism of BS-Z15 secondary metabolites in regulating immune function in mice

This study demonstrated that BS-Z15 secondary metabolites enhanced immune function in mice. After oral administration of BS-Z15 secondary metabolites at a dose of 90 mg/kg, mice showed significant enhancement of innate, cellular and humoral immune functions, as shown in Fig. 8 in this study. Analysis of blood transcriptome data of mice by RNA-seq technique revealed that BS-Z15 secondary metabolite increased the activity and function of NK cells and monocytes-macrophages, mainly through TLR and TNF signaling pathways. The upregulation of C1qb and C4b activates the complement pathway, enhances innate immune function in mice and significantly increase the levels of IgG, IgM IL-6 and IFN-γ in plasma. This treatment also promoted the secretion of cytokines in *vivo*, upregulated the expression of genes encoding Tetracyclin Resistant (TCR) and RFX5, and promote the binding of TCR and major histocompatibility complex II (MHC II) in mice, thereby sustaining a high proportion of functionally activated immune cells, promoting T cell transformation and increasing the content of B lymphocytes to improve the adaptive immune function in mice.

Blood transcriptomic analysis revealed that the pathways of BS-Z15 secondary metabolites affecting immune function in mice were mainly enriched in the TLR signaling pathway, response to interferon-β, tumor necrosis factor signaling pathway, systemic lupus erythematosus, and primary immunodeficiency. TLRs are a type of PRR that recognize pathogen-associated molecular patterns (PAMPs) in different microorganisms and act as a bridge between the innate and adaptive immune systems [27], activating multiple signaling pathways in immune cells and thus, providing a defense mechanism against pathogens [5]. TLR2-TLR1 or TLR2-TLR6 heterodimers recognize different pathogen-expressed PAMPs including lipoproteins, peptidoglycans and yeast glycans [28]. Probiotic *Bacillus amyloliquefaciens* SC06 can increase immune function by activating the TLRs signaling pathway [29], and isoleucine can regulate host innate and adaptive immunity via increasing the expression of β-defensins to activate the TLR/NF-kB signaling pathway [30]. Since cyclic lipopeptides are major components of the BS-Z15 secondary metabolites, it can be speculated that these lipopeptides are recognized by TLR2-TLR1 or TLR2-TLR6 heterodimers, but this needs to be confirmed by further studies. BS-Z15 secondary metabolites lead to activation of TLR signalling pathways, up-regulated expression of IKKα and MMK7, which trigger the mitogen-activated protein kinase (MAPK) and NF-κB signalling pathways and expression of activated transcription factor activator protein-1 (AP-1), and up-regulate expression of Ccl3, the gene encoding macrophage inflammatory protein 1-alpha. Genistein (GEN) improve immune function of broiler chicks via activating Toll-like receptor signaling pathways and MAPK cascade signaling pathways, and significantly increasing antigen processing and presentation, macrophage activation, B lymphocyte, NK cell and helper T cell proliferation and CD4 + T lymphocyte differentiation [31].

BS-Z15 secondary metabolite treatment up-regulated IKKα binds to interferon response factors, leading to the release of IFN-α and IFN-β [32], and activation of the response to IFN-β in mice. IFN-β enhances the immune response, activating the innate and adaptive immune responses and inhibiting viral infection and replication [33]. AP-1, a key downstream target of the MAPK signaling pathway, regulates the expression of many cytokines including TNFα, IL-6, IL1 and plays a key role in the innate immune response [34]. Upregulation of CCL3 expression enhances the release of innate immune cells (e.g., NK cells and DCs) from bone marrow into the blood, mobilizes NK cells to produce IFNγ, and increases the level of IFNγ in the blood of mice, which is consistent with the significant increase in the level of IFN-γ in the blood of mice in the BS-Z15 secondary metabolite treatment group. In addition, NK cells perform immunosurveillance functions in the absence of antigenic stimuli and antibodies, directly lysing and removing tumor cells and virus-infected cells [35].CCL3 also initiates the differentiation of progenitor T cells to CD4^+^ T cells and CD8^+^ T cells [36], and the recruitment of DCs and T cells increases the chance of encounters between antigen-specific T cells and professional antigen-presenting cells (pAPC), thereby regulating the adaptive immune response in mice [37].

TNF is an inflammatory cytokine produced mainly by monocytes, macrophages and NK cells, and functions by activating two different receptors, TNFR1 and TNFR2, to induce lymphocyte and leukocyte activation and migration and inflammatory responses [38]. Following infection, monocytes migrate from the blood to infected or injured tissues in a process that is regulated by platelets, where they differentiate into macrophages. Macrophages recognize foreign bodies and fight infection directly through phagocytosis or a pro-inflammatory response, and subsequently control the infection through an anti-inflammatory response, essential for cellular innate immunity [39, 40]. CMP can improve the immunity of mice by regulating TNF signaling pathway and increasing the number of white blood cells, the degree of delayed allergy and the content of hemolysin in the serum [41].BS-Z15 secondary metabolite treatment increased the number of platelets and monocytes in the blood of mice, significantly enhanced the function of monocytes-macrophages and NK cells, increased the level of tumor necrosis factor and activated the tumor necrosis factor signaling pathway to regulate the intrinsic immune function of mice.

The silencer of death domain (SODD) is a key negative regulatory protein in this signaling pathway in the TNFR1-mediated pathway and contributes to the sensitivity of TNF binding to receptors [42, 43]. In this study, Bag4, the gene that encodes SODD, was significantly downregulated, while the expression of Gm17809, the gene encoding IKKα, and Gm49320, the gene encoding MMK7, were upregulated [44]. MKK7, which plays a key role in the activation of the nuclear factor kappa-B (NF-κB) pathway, is a key kinase in the activation of JNK by TNFα and in the activation of macrophages [45]. The BS-Z15 secondary metabolite was shown to activate the downstream NF-κB and MAPK signaling pathways via a cascade reaction, upregulate the expression of Fos and Jun genes, activate the AP-1 transcription factor, and influence the upregulation of AP-1 downstream of the TLR signaling pathway to control immune functions. Daidzein (DA) treatment upregulated the expression of Fos, and Jun in chickens and affected the expression of AP-1 to regulate MAPK signaling, Toll-like receptor signaling, and related mRNA expression. And it also enhanced macrophage activity, increased the numbers of blood mononuclear cells (157), and the IgA and IgG concentrations, antibody titers, antioxidant capacity and B lymphocyte differentiation of broilers were increased [46]. Thus, it can be hypothesized that the secondary metabolites of BS-Z15 enhance innate immune function in mice through activation of the TNF signaling pathway regulation of the expression levels of cytokines involved in adaptive immunity and adaptive immune function in mice, which can improve the body’s ability to respond to bacterial and viral infections.

Defects in C1q and C4 cause Systemic Lupus Erythematosus (SLE). However, in the present study, C1q and C4 expression was upregulated, indicating that treatment of mice with BS-Z15 secondary metabolites did not cause SLE [47, 48]. Complement is an important component of the innate immune system and is involved in regulation of the adaptive immune system, in addition to its key role in fighting infection via the innate immune system [49]. C1q, which functions as a PRR of the innate system, recognizes many auto-, non-auto- and altered self-ligands [50]. This interaction enhances the recognition and phagocytosis of targets suboptimally conditioned with antibodies or complement through conditioning effects and upregulation of phagocytosis mechanisms in macrophages. It also promotes host defense in primary immune responses in immunocompromised individuals [51] and maintains immune tolerance to prevent symptoms of immune overload in the body [47]. C4 is required for normal T cell activation, proliferation and survival, and T cells from C4 knockout mice show reduced activation and decreased proliferation, survival and IFNγ production following activation [49]. Chitosan-Zn chelate could increase serum complement 3, and complement 4 levels and improve immune function in weaned piglets [52]. The T cell antigen receptor (TCR) is expressed on T cells and coordinates the adaptive immune response [53] to peptide antigens presented in the context of major histocompatibility complex (MHC) molecules, which is the first step in T cell activation. In organisms with a high TCR co-expression profile, the activity of signaling pathways such as lymphocyte activation and proliferation is significantly upregulated, which activates and regulates immune cells and mediates the activation, proliferation and differentiation of T and B cells. This ultimately serves to activate the inflammatory response of the organism to clear the infection [54]. RFX5 is the key MHCII gene transcriptional regulator.

BS-Z15 secondary metabolites enhance the host’s sensitivity to pathogen recognition through upregulation of C1q, directly enhancing phagocytosis or activating the classical complement pathway, and promoting T cell proliferation and activation to enhance the host’s defense. C1q also maintains the dynamic balance of the immune response. And the high expression of TCR and RFX5 in the blood transcriptome of mice in this study indicates that the secondary metabolites of BS-Z15 promote the binding of TCR and MHC II in mice, thereby increasing the number of mature CD4^+^ T cells (Th0) in the body, and sustaining a high proportion of functionally activated immune cells with enhanced potential to bind cytokines and chemokines and activate cellular and humoral immune responses to pathogen invasion the body. Serum IgM and IgG, complement C3, C4 and IL-6 levels were significantly increased with Chinese yam polysaccharides (CYP) treatment [55]. Spirulina increases the IgM level and improves immune function after chemotherapy in patients with malignant tumors [56]. The cellular immune function of mice was activated as evidenced by the significant increase in T-cell conversion rate and swelling of the left hind foot and metatarsal area of mice in the high dose group; the ability of antibody-forming cells to produce antibodies was significantly increased, and the hemolytic value of plasma and the IgG and IgM contents of mice in the high dose group were significantly increased, which proved that the cellular immune function of mice was activated.

Extracellular histones are associated with the stimulation of strong inflammatory responses and inflammatory damage processes that are harmful to the organism and the immune system [57, 58]. CD40L can synergize with antigens to regulate the adaptive immune function of B cells [59]. BS-Z15 secondary metabolite treatment was found to be associated with activation of B cells and humoral immunity mainly through TCR binding to MHC II. These interaction represent immune synapses, which promote stronger T and B cell binding and confine cytokines secreted by Th cells to synaptic sites, thereby efficiently promoting further B cell proliferation, class switching, affinity maturation, antibody production and differentiation into plasma cells or memory B cells. Type I interferon, which also induces B cell activation and IL7, which is involved in B cell development. were both upregulated in mice following BS-Z15 secondary metabolite treatment, indicating its involvement in regulating the adaptive immune function of B cells. The down-regulation of histone H4 and CD40L in mice did not inhibit the activity of B cells and humoral immunity, but rather inhibited the production of a strong immune response and coordinated its level, The study showed that SARS-CoV-2 infection activates a cytokine storm through a CD40-CD40l dependent pathway [60], so down-regulation of CD40lg may manage the cytokine storm triggered by viral infection, which is beneficial to tolerance and protection against uncontrolled immune responses.

The dose of treatment are directly related to the efficacy of the drug. In a previous study [61], 90 mg/kg BS-Z15 secondary metabolite significantly reduced the level of lipid peroxidation and significantly increased the ability of mice to scavenge oxygen radicals, thus increasing the total antioxidant capacity in mice. In combination with the results of this study, 30 mg/kg had a modulating effect on immune function but did not have significant efficacy in the treatment of fungal infections in animals. 90 mg/kg of BS-Z15 secondary metabolite antagonized Candida albicans in the treatment of infected animals and systematically activated both innate and adaptive immune functions in mice against fungal infections. Therefore, the recommended therapeutic dose of BS-Z15 secondary metabolite is 60–90 mg/kg in patients with fungal infections or immunocompromised by clinical treatment, while 30 mg/kg can be used for prophylaxis, consolidation of efficacy or treatment of thrombocytopenia. The results of this study provide predictive evidence for the development of BS-Z15 secondary metabolites into immune enhancing drugs with clinical applications.

### Electronic supplementary material

Below is the link to the electronic supplementary material.

Supplementary Material 1

Supplementary Material 2

FIGURE S1 DEGs were analyzed by GO annotation.GO annotation analysis of DEGs. The horizontal axis represents number of genes, and the vertical axis represents the GO term. Red represents cellular component (CC), blue represents molecular function (MF), green represents biological process (BP). FIGURE S2 DEGs were analyzed by KEGG annotation.KEGG annotation analysis of DEGs. The horizontal axis represents the KEGG pathway, and the vertical axis represents number of genes. Red represents metabolism, blue represents genetic information processing, green represents environment information processing, dark grey represents cellular processes, orange represents organismal system, light grey represents human disease.

Table S1 Primers used for qPCR.

## Data Availability

The datasets generated for this study can be found in the BioProject accession: PRJNA899087, https://www.ncbi.nlm.nih.gov/bioproject/PRJNA899087/.
