# Correction to: Effect of bacillus subtilis strain Z15 secondary metabolites on immune function in mice

**DOI:** 10.1186/s12864-023-09641-6

**Published:** 2023-09-07

**Authors:** Xi-Yuan Cao, Reyihanguli Aimaier, Jun Yang, Jing Yang, Zhong-Yi Chen, Jing-Jing Zhao, Li Yin, Qi Zhang, Jia You, Hui Zhang, Hao-Ran Li, Jia-Yi Chen, Qing-Chen Mao, Li-Ping Yang, Fei Yu, He-Ping Zhao, Hui-Xin Zhao

**Affiliations:** 1 Xinjiang Key Laboratory of Special Species Conservation and Regulatory Biology, College of Life Science, Xinjiang Normal University, Urumqi, China; 2 Beijing Key Laboratory of Gene Resource and Molecular Development, College of Life Sciences, Beijing Normal University, Beijing, China

**Correction:** ***BMC Genomics*** **24, 273 (2023).**

10.1186/s12864-023-09313-5

Following publication of the original article [1] it was reported that there was a missing designation of co-first authorship for Xi-Yuan Cao, Reyihanguli Aimaier, Jun Yang, and Jing Yang. The correct authorship is given in this Correction article and the original article has been updated.
