# Supplementary figures and images for: Effect of bacillus subtilis strain Z15 secondary metabolites on immune function in mice
Source: BMC Genomics. 2023 May 19;24:273. doi: 10.1186/s12864-023-09313-5 (PMC10198031; doi:10.1186/s12864-023-09313-5)

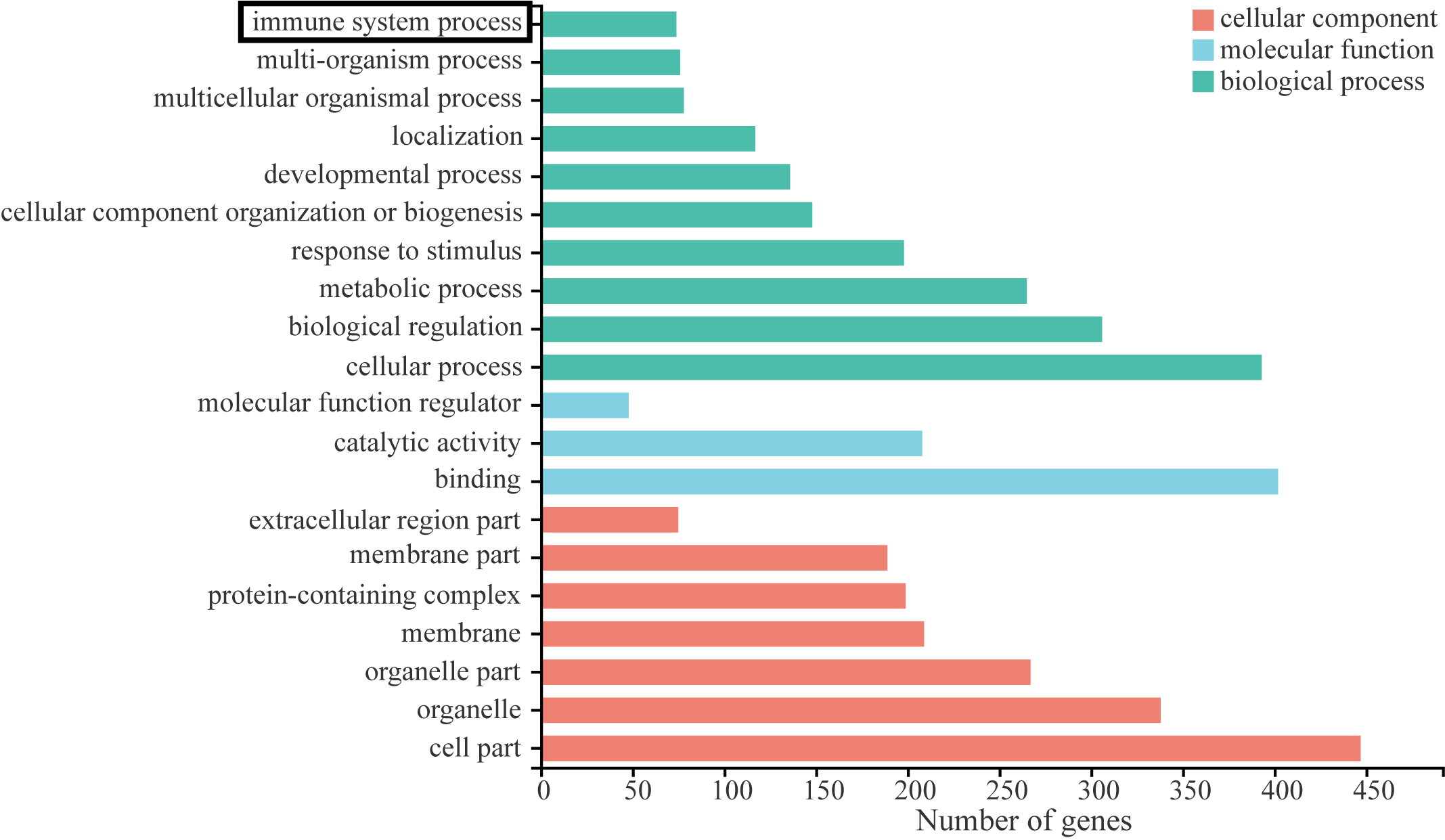

Supplement: Supplementary file 1 — Supplementary Material 1 [file 12864_2023_9313_MOESM1_ESM.png]

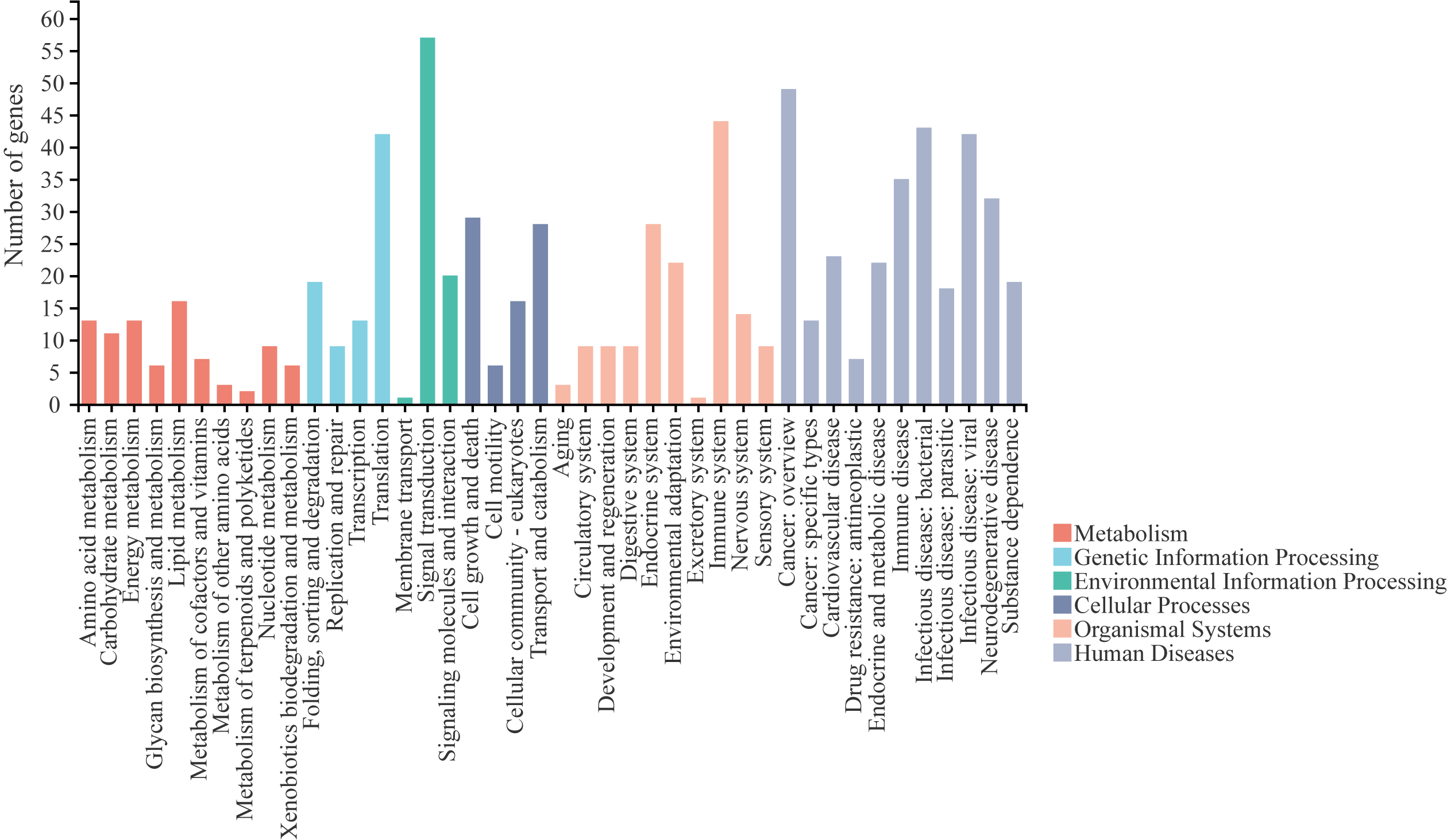

Supplement: Supplementary file 2 — Supplementary Material 2 [file 12864_2023_9313_MOESM2_ESM.png]
