# Supplementary material for: Effect of bacillus subtilis strain Z15 secondary metabolites on immune function in mice
Source: BMC Genomics. 2023 May 19;24:273. doi: 10.1186/s12864-023-09313-5 (PMC10198031; doi:10.1186/s12864-023-09313-5)
Supplement: Supplementary file 4 — Table S1 Primers used for qPCR. [file 12864_2023_9313_MOESM4_ESM.docx]

Table S1 Primers used for qPCR

| Gene Name | Forward primer 5’-3’ | Reverse primer 5’-3’ |
| --- | --- | --- |
| Trav16d-dv11 | ACGGTGACAATGGACTGTGT | CTGTGTGGCGGTGATGATGA |
| Map2k7 | CAGCGTTATCAGGCAGAA | ACTACATCCAGGTCCATCA |
| Fos | GCTTTCCCCAAACTTCGACC | ATCTGCGCAAAAGTCCTGTG |
| Lrrc32 | GCCCTGTAGGACGGTGAACAA | CAGTTGGTTCCCGGACAAGTA |
| Ccl3 | TGCCAAGTAGCCACATCGAG | GAGATGGGGGTTGAGGAACG |
| Ccr5 | AGACATCCGTTCCCCCTACA | GCAGGGTGCTGACATACCAT |
| β-actin | ACCCTAAGGCCAACCGTGAA | ATGGCGTGAGGGAGAGCATA |

FIGURE S1 DEGs were analyzed by GO annotation.GO annotation analysis of DEGs. The horizontal axis represents number of genes, and the vertical axis represents the GO term. Red represents cellular component (CC), blue represents molecular function (MF), green represents biological process (BP).

FIGURE S2 DEGs were analyzed by KEGG annotation.KEGG annotation analysis of DEGs. The horizontal axis represents the KEGG pathway, and the vertical axis represents number of genes. Red represents metabolism, blue represents genetic information processing, green represents environment information processing, dark grey represents cellular processes, orange represents organismal system, light grey represents human disease.
